# Case Report: Use of Liposomal Amphotericin B in Low Doses in Patients With Visceral Leishmaniasis

**DOI:** 10.3389/fmed.2021.766400

**Published:** 2021-11-17

**Authors:** Danfeng Ren, Wenya Cao, Xiaojing Liu, Qunying Han, Wanhu Fan, Guoliang Li, Han Xia, Xi Zhang

**Affiliations:** ^1^Department of Infectious Diseases, The First Affiliated Hospital of Xi'an Jiaotong University, Xi'an, China; ^2^Dialysis Department of Nephrology Hospital, The First Affiliated Hospital of Xi'an Jiaotong University, Xi'an, China; ^3^Department of Cardiovascular Medicine, The First Affiliated Hospital of Xi'an Jiaotong University, Xi'an, China; ^4^Hugobiotech Co., Ltd., Beijing, China

**Keywords:** visceral leishmaniasis (VL), case series, amphotericin B (AMB), liposomal amphotericin B (L-AmB), the low-dose L-AmB therapy

## Abstract

**Background:** No consensus has been reached regarding the optimal therapy for visceral leishmaniasis (VL), which affects ~12 million people worldwide.

**Case Presentation:** This report described four cases of VL encountered in the First Affiliated Hospital of Xi'an Jiaotong University between October 2019 and December 2020. Of the four patients, one patient experienced relapse after antimonial treatment, and the remaining patients had primary VL (including one patient with impaired kidney function and one patient with hemophagocytic syndrome). All patients received a novel treatment protocol, namely the low-dose L-AmB therapy, which was characterized by a low initial dose, cautious dose escalation, and low-dose therapy as maintenance. All patients were cured without severe complications, and there was no further recurrence during follow-up.

**Conclusions:** This case series demonstrated the safety and efficacy of the low-dose L-AmB therapy for VL patients, providing novel treatment protocol for the VL.

## Background

Visceral leishmaniasis (VL) (also known as kala-azar) is a chronic endemic infectious disease caused by the *Leishmania donovani*, which is most transmitted through sandly bites. Patients with classic VL present with a characteristic pentad of prolonged irregular fever, weight loss, hepatosplenomegaly (especially splenomegaly), pancytopenia, and hyperimmunoglobulinemia (1, 2). VL afflicts 1.5–2 million people each year, and more than 380 million are at risk in 98 subtropical and tropical countries worldwide (3, 4). Over 90% of the patients live in India, Brazil, Sudan, Bangladesh, Nepal, and Ethiopia (4–6). ~ 400 cases of VL are reported in China each year, which mainly occur in six western provinces (Xinjiang, Gansu, Sichuan, Shanxi, Shaanxi and Inner Mongolia). In Shaanxi province, VL is mainly prevalent in Yichuan county and Hancheng City which are located in the junction of the Loess Plateau and the Guanzhong Plain, especially in Hancheng. Recently, the warming climate and the intensified population movement has caused expanding endemic zones and widespread morbidity, which make VL an acute threat to public health and social economy (7–9).

Existing therapeutic options for VL have several limitations. Pentavalent antimonials were the first and the most common drugs used to treat VL (10, 11). Over time, escalating drug resistance exhibited by *Leishmania* parasites had gradually led to the withdrawal of antimony drugs from therapeutic regimens in some regions. Amphotericin B (AMB) and its liposomal formulation (L-AmB) have been gradually employed in many Western countries because of their efficacy (5, 12, 13). However, inevitable severe side effects limit their use (14). Therefore, effective and safe treatments for VL are urgently needed. We proposed a novel approach called the low-dose L-AmB therapy as a method for treating VL. The treatment starts with an average L-AmB dose of 0.1–0.15mg/(kg·day) for a few days. Then, the dose is increased by 0.2 mg/(kg·day) every 1–2 days. Finally, a low dose of L-AmB [0.6–1.2mg/(kg·day)] is applied for maintenance therapy to maximize efficacy and minimize toxicity until the cumulative dose recommended by the World Health Organization (WHO) is achieved (15). A low initial dose and gradual dose escalation help clinicians to adjust the dosage timely according to the patients' condition, which has a great utility for reducing complications. This report describes our experience using the low-dose L-AmB therapy in four patients with VL to provide a basis for selecting safe and effective therapeutic options in VL treatment.

## Case Presentation

### Case 1

**Figure 1:**
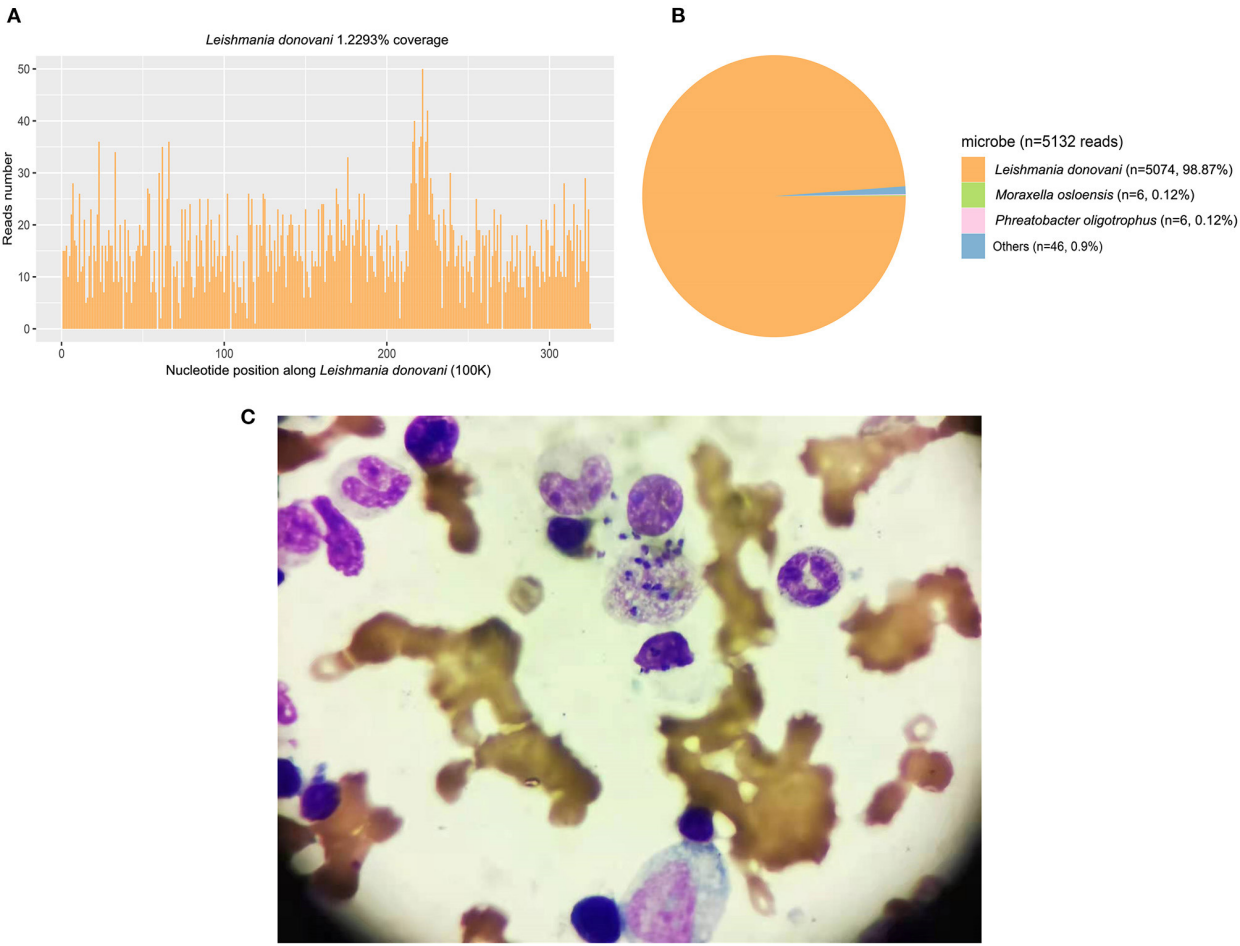
The mNGS and bone marrow result detected in Case 1. **(A)** The genome coverage of the detected reads of *Leishmania* shedunii. **(B)** The abundance of detected microbe and their reads numbers. A total of 5074 specific reads (98.87%) of *Leishmania* were detected in the blood sample. **(C)** The examination of a bone marrow aspirate revealed amastigotes.

A 60-year-old previously healthy male farmer presented to the First Affiliated Hospital of Xi'an Jiaotong University with a 6-month history of intermittent fever. Five months ago, he was diagnosed with VL and treated with sodium antimony gluconate (SAG) (0.6 g/d) for 6 days. The patients' condition improved, and he was discharged from the hospital. However, he was readmitted to our hospital because of recrudesce fever. He had a history of working in coalmines and current hypertension treated with oral therapy. There was no complication related to hypertension. Medical, surgical, pharmacological and family histories were not significant. His body weight was 70 kg. Physical examination revealed splenomegaly, no superficial lymphadenopathy, and no edema of the lower limbs. Blood cultures as well as tests for bacteria, fungi, tuberculosis, parasites, and brucellosis agglutination were all negative. His laboratory findings included pancytopenia with a C-reactive protein (CRP) level of 82.8 mg/L and procalcitonin (PCT) level of 0.88 ng/mL. The patient's liver function and coagulation function were abnormal. Computed tomography of the abdomen confirmed the presence of splenomegaly. Examination of bone marrow aspirate revealed amastigotes, which is the typical sign of leishmaniasis. PACE-seq metagenomic next-generation sequencing (mNGS) (Hugobiotech, Beijing, China) detected 5,074 specific reads of *Leishmania* in the blood sample, also indicating leishmaniasis (Figure 1). The polymerase chain reaction (PCR) test of the bone marrow aspirate confirmed the diagnosis. He was thus diagnosed with recurrent VL.

**Figure 2:**
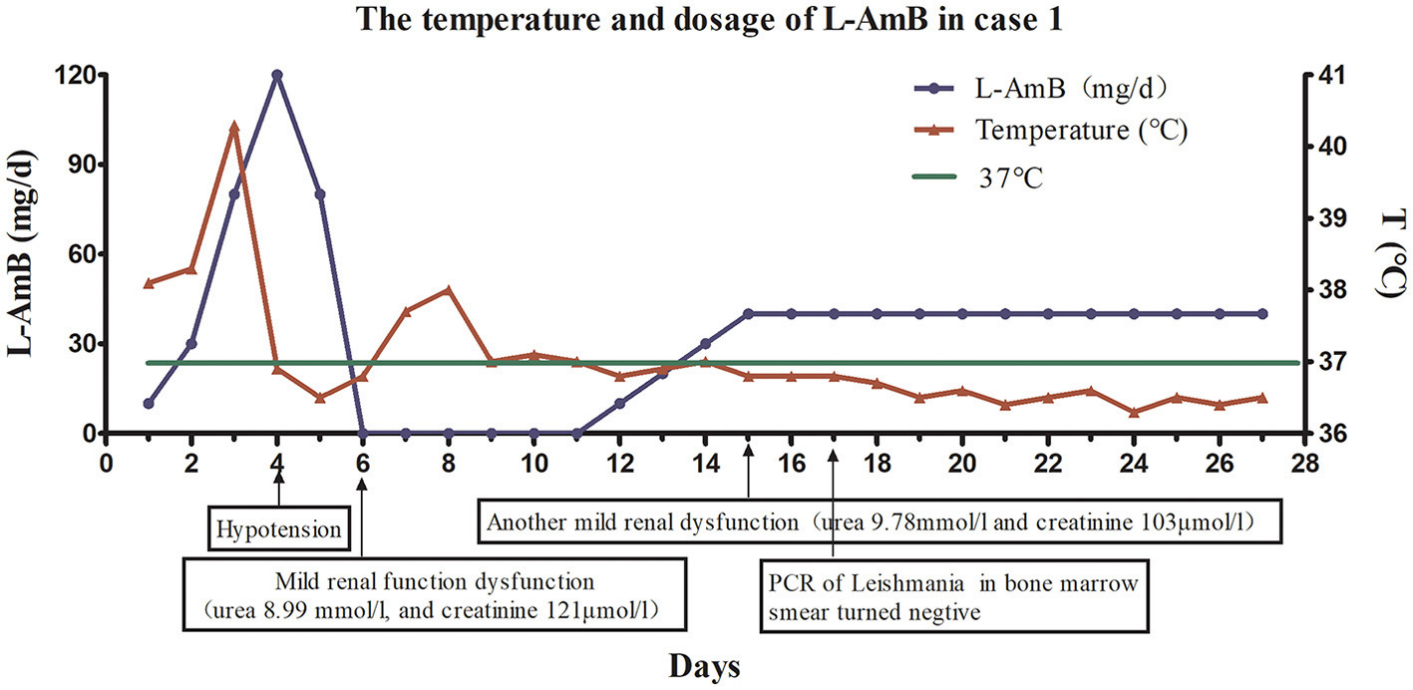
The dose of liposomal amphotericin B (L-AmB) and changes of temperature in Case 1. The initial dosage of L-AmB in the first patient was 10mg/day and the patient's temperature decreased as the dose of L-AmB was escalated. Dose escalation was performed as follows: 10 mg on day 1, 30 mg on day 2, 80 mg on day 3, and 120 mg on day 4. However, the appearance of hypotension on day 4 of the treatment protocol forced the dose reduction on day 5 (80 mg/d). There was mild renal impairment on day 6. Therefore, treatment was interrupted on days 6–11, and his renal function gradually recovered during treatment cessation. Treatment was restarted at a dose of 10 mg/kg from day 12, and the dose was gradually increased at a rate of 10 mg/(kg·day). The dose was increased to 40 mg on day 15. Since the patient's renal function was still slightly abnormal at day 15, the dose of L-AmB was not increased, but maintained at 40 mg during the next 12 days (day 16–27). Finally, we stopped the medication until the accumulating dose reached 890 mg(12.6 mg/kg) on the day 27. Polymerase chain reaction of *Leishmania* in a bone marrow smear was negative on day 17.

Because the patient experienced relapse after treatment with antimonials, he received L-AmB. The starting dose of L-AmB was 10 mg/day (~0.15 mg/kg). Then dose escalation of the L-AmB for the patient is presented in Figure 2. On the 17th day of the treatment protocol when the accumulated dose reached 500 mg (10 mg/kg), PCR of the bone marrow aspirate of *Leishmania* was negative. The patient's blood cell counts (red blood cells, white blood cells, and platelets) increased gradually, and his spleen size decreased gradually. During the treatment, patient's liver function remained normal. There was no recurrence of VL at the 1-year follow-up visit. The patient's temperature and L-AmB dose were presented in Figure 2, and his basic information was summarized in Table 1.

**Table 1:** Basic patient information and examination results before and after treatment.

	**Case1**	**Case 2**	**Case 3**	**Case 4**
Age/ Sex	60/M	53/F	56/F	46/M
Epidemiological history	Travel history to endemic areas	Travel history to endemic areas	Travel history to endemic areas	Travel history to endemic areas
Initial treatment / Relapse	relapse	Initial treatment	Initial treatment	Initial treatment
Complications	-	-	AKI	HLH
Pre-treatment				
Blood routine test				
White blood cell counts [(3.5-9.5) × 10^9^/L]	1.19	1.71	1.06	1.13
Platelet count [(125-350) × 10^9^/L]	42	31	8	32
Red blood cell count [(4.3-5.8) × 10^12^/L]	2.5	3.2	2.68	2.5
Hemoglobin concentration [(130-175)g/L]	69	73	55	70
Spleen size				
Line I (cm)	12.5	9	9	9
Line II (cm)	16	9	14	8
Line III (cm)	−3	−4.5	2	−5.5
Bone marrow examination	PCR (+); A bone marrow aspiration revealed Leishmania	PCR (+)	PCR (+)	PCR (+)
Post-treatment/before discharge				
Blood routine test				
White blood cell counts [(3.5-9.5) × 10^9^/L]	2.39	3.02	1.85	3
Platelet count [(125-350) × 10^9^/L]	112	134	27	222
Red blood cell count [(4.3-5.8) × 10^12^/L]	3.89	4.58	3.04	3.38
Hemoglobin concentration [(130-175)g/L]	108	121	75	96
Spleen size				
Line I (cm)	8	4	7	4
Line II (cm)	8	1	10	1
Line III (cm)	−8	−7.5	0	−8.5
Bone marrow examination	PCR (-)	PCR (-)	PCR (-)	PCR (-)
Follow-up after discharge	1year of follow-up	1year of follow-up	6 months of follow-up	6 months of follow-up
Blood routine test				
White blood cell counts [(3.5-9.5) × 10^9^/L)]	5.09	7.05	2.79	8.02
Platelet count [(125-350) × 10^9^/L]	189	251	66	182
Red blood cell count (4.3-5.8) × 10^12^/L]	4.89	4.78	2.91	4.97
Hemoglobin concentration [(130-175)g/L]	140	132	90	155
Spleen size	Untouched below the costal margin	Untouched below the costal margin	3 cm below the costal margin	Untouched below the costal margin
Bone marrow examination	PCR (-)	PCR (-)	PCR (-)	PCR (-)

*AKI, acute kidney impairment; HLH, hemophagocytic lymphohistiocytosis; PCR, polymerase chain reaction; PCR (+), PCR for the detection of Leishmania was positive; PCR (−), PCR for the detection of Leishmania was negative; Spleen line I, the distance between the midline of the left clavicle and the costal margin to the lower margin of the spleen; Spleen line II, the distance between the midline of the left clavicle and the intercostal margin to the farthest point of the spleen; Spleen line III, the distance between the right margin of the spleen and the anterior median line*.

*The spleen crosses the anterior median line to the right, which was measured as a positive value, and vice versa*.

### Case 2

**Figure 3:**
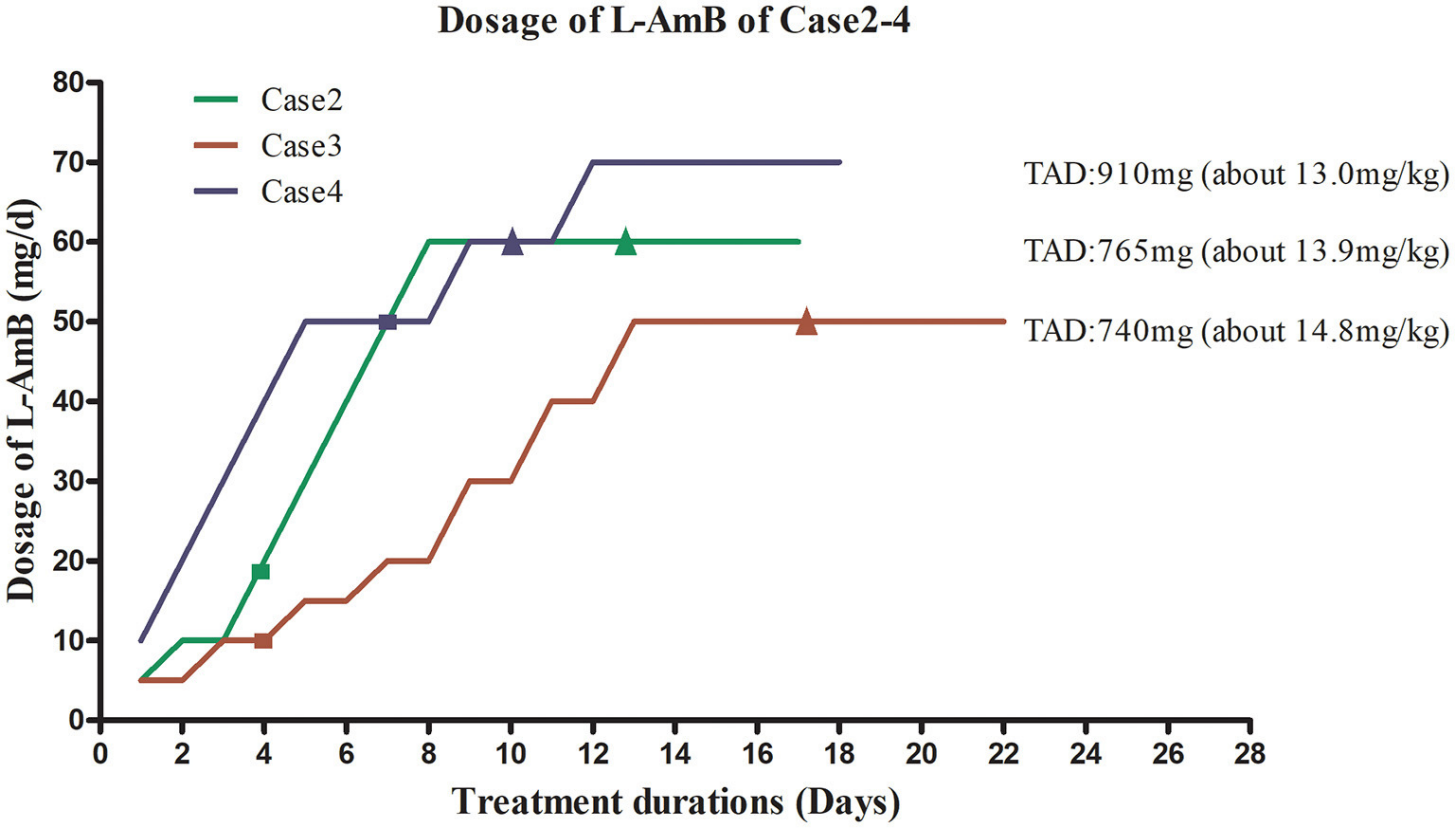
Liposomal amphotericin B (L-AmB) administration protocol and temperature changes in Cases 2–4. TAD, total accumulated dosage. ■ Normalization of body temperature. ▴ Negative PCR result for bone marrow aspirate. To avoid complications associated with adverse drug reactions, the dosing regimens of L-AmB in case 2–4 were similar to those used for case 1, starting with a low dose that was gradually increased until the maximum tolerated dose was reached. This dosage was used for maintenance therapy. The initial dose for case 2 was 5 mg (~0.1 mg/kg) on day 1. The dose was increased to 10 mg on day 2–3, and no complications occurred. Therefore, the patient received L-AmB in 10 mg dose increments up to a dose of 60 mg (~1.2 mg/kg) on day 8. This dosage (60mg) was used for maintenance therapy until the cumulative dose of L-AmB reached 765 mg (13.9 mg/kg) on day 17. Her body temperature returned to normal on day 4. Polymerase chain reaction (PCR) for *Leishmania* in bone marrow aspirate was negative on day 12, when the dose of L-AmB was 405 mg. The trapezoidal regimen of L-AmB in case 3 was initiated at a dose of 5 mg (~0.1 mg/kg). The dosage of L-AmB was increased by 5 mg every 2 days as tolerated from day 1 to day 8 and increased by 10 mg every 2 days from day 9 to day 13 to 50 mg (~1.0 mg/kg), which was administrated as maintenance therapy on days 14–22. Treatment was stopped when the cumulative dose of L-AmB reached 740 mg(14.8 mg/kg) on day 22 of protocol. The patient's body temperature had returned to normal on day 4. PCR for *Leishmania* in bone marrow aspirate was negative on day 15, when the cumulative dose of L-AmB was 400 mg. In case 4, L-AmB treatment was initiated at a dose of 10 mg and increased in increments of 10 mg (~0.14 mg/kg) daily, reaching 70 mg (~1.0mg/kg) on day 7 of the protocol. To avoid complications, instead of further increasing the L-AmB dose, we used a dose of 70 mg as maintenance therapy until the cumulative dose reached 910 mg (13 mg/kg) on day 16. His body temperature returned to normal on day 7. PCR for *Leishmania* in bone marrow aspirate was negative on day 9, when the cumulative dose of L-AmB was 420 mg.

A 53-year-old woman with no significant underlying medical history was admitted to our hospital with a 1-month history of intermittent fever. She ever picked *Zanthoxylum* seeds in Hancheng, China 8 years ago, and she had a history of mosquito bites. She had a history of esophageal cancer and received surgical treatment one and a half years ago, and there was no recurrence after operation. Past medical, past surgical, drug and family histories were not significant. Her body weight was 55 kg. Physical examination revealed splenomegaly and axillary and inguinal lymphadenopathy. Laboratory tests revealed high levels of CRP (97.3 mg/L; reference value, <10.0 mg/L), as well as elevated liver enzyme levels, abnormal coagulation, and pancytopenia. Her PCT level was 1.24 ng/mL. Antibodies against *Leishmania* were detected in her blood. A positive PCR result for *Leishmania* in bone marrow aspirate confirmed the diagnosis of VL. She received prompt treatment with L-AmB at an initial dose of 5 mg (~0.10 mg/kg) on the first day. The L-AmB protocol for the patient is presented in Figure 3. During L-AmB dose escalation, laboratory tests of renal functions and electrolytes uncovered no particular abnormality during treatment. Routine blood parameters gradually normalized during therapy, and her spleen size gradually decreased. No VL recurrence was observed at the 1-year follow-up visit.

### Case 3

A 56-year-old woman presented with fever. Her symptoms weren't improved after 1 week of anti-infective treatment in a local hospital. Similar to case 2, she also had picked *Zanthoxylum* seeds in Hancheng 1 years ago, and she had a history of mosquito bites. She was healthy in the past. Her body weight was 50 kg. Physical examination revealed splenomegaly, mild lower extremity edema, and multiple skin petechiae. Blood cultures as well as tests for bacteria, fungi, tuberculosis, parasites, and brucellosis agglutination were all negative. The patient's laboratory results revealed pancytopenia and elevated liver enzyme levels (aspartate aminotransferase, 121 U/L; alanine aminotransferase, 36 U/L). Her PCT, CRP, and creatinine levels were 5.67 ng/mL, 89.7 mg/L, and 641 μmol/L, respectively. Computed tomography of the abdomen revealed homogeneous splenomegaly. Her bone marrow smear was negative for *Leishmania*. Conversely, antibodies against *Leishmania* were detected in peripheral blood, and PCR was positive for *Leishmania*. The patient was diagnosed with VL combined with acute renal impairment.

Continuous renal replacement therapy (CRRT) was administered on days 2, 4, and 6 after admission, and this treatment resulted in significantly diminished creatinine levels. To avoid the aggravation of pre-existing renal injury, the low-dose therapy of L-AmB was initiated with a starting dose of 5mg (~ 0.10 mg/kg) on days 9–10 after admission. The L-AmB administration protocol is presented in Figure 3. During therapy, an increase of plasma creatinine levels was observed, peaking at 178 μmol/L on day 4 of treatment. Her creatinine level subsequently declined to 148 μmol/L on day 8 in the absence of CRRT. Thus, slow dose escalation appeared to prevent the further deterioration of renal function. The patient's creatinine further declined during maintenance therapy. Routine blood testing revealed gradual increase of red blood cell, white blood cell, and platelet counts. Her spleen size was gradually decreased, and her kidney function was gradually improved by therapy. The patient's liver function also gradually returned to normal during treatment. No relapse was observed during the 3- and 6-month follow-up visit. In addition, we observed complete recovery of her renal function at 6 months after discharge (urea, 5.66 mmol/L; creatinine, 75 umol/L).

### Case 4

A 46-year-old man presented with a 3-week history of fever and pancytopenia. He had worked in coalmines in Hebei Province, Henan Province, and Shanxi Province for more than 10 years. There was negative past medical and past surgical history. His body weight was 70 kg. Physical examination suggested sporadic hemorrhagic spots on his skin of chest and back, palpable right sided cervical lymphadenopathy, and splenomegaly. Blood cultures as well as tests for bacteria, fungi, tuberculosis, parasites, and brucellosis agglutination were all negative. The laboratory examination revealed pancytopenia, coagulation abnormalities, and elevated liver enzyme levels. The patient's PCT and CRP levels were 0.99 ng/mL and 73.4 mg/L, respectively. Bone marrow biopsy disclosed hypocellular marrow with trilineage hematopoiesis and macrophages with microorganisms. The aspirate prominently contained hemophagocytic macrophages. Additional investigations identified elevated ferritin and soluble interleukin-2 receptor (sCD25) levels and absent natural killer (NK)-cell activity, supportive of hemophagocytic lymphohistiocytosis (HLH). Antibodies against *Leishmania* in peripheral blood and PCR were positive, confirming the diagnosis of VL. He was received intravenous gammaglobulin. The L-AmB protocol followed that presented in Figure 3. The initial dose was 10 mg (~ 0.14 mg/kg), and the maintenance dose was 70 mg (~1.0 mg/kg). The total accumulative dosage was 910 mg (13 mg/kg), which was close to the WHO-recommended dosage. The patient's liver enzymes (AST and ALT) were mild abnormal before the treatment. He received the low-dose L-AmB therapy in addition to liver-protecting treatment. His liver function gradually recovered and kidney function remained normal during the treatment. No complications occurred during treatment excluding hypokalaemia, which was corrected via potassium supplementation. The patient's blood routine parameters gradually normalized and spleen size gradually decreased during therapy. No VL recurrence was observed at the 6-month follow-up visit.

## Discussion and Conclusions

Leishmaniasis, a global disease of poverty, mainly occurs in remote, underdeveloped areas where access to health-care facilities is limited or non-existent (16, 17). Because of its high biological heterogeneity, no treatment option has been found to be universally effective. Pentavalent antimony has been implemented into VL treatment since 1916, and this regimen has remained reliable after more than a century of clinical practice. Antimonial drugs were initially the first-line drugs recommended by WHO, but they have been gradually replaced by L-AmB because of multiple toxicities and inefficacy associated with resistance (5, 15, 17, 18).

The polyene anti-fungal agents AMB and L-AmB exert their fungicidal effects by binding to sterols, i.e., ergosterol of *Leishmania* and cholesterol of host macrophages, leading to the formation of aqueous pores in leishmanial promastigote cell membrane and osmotic changes that result in cell lysis and markedly inhibit the ability of *L. donovani* promastigotes to bind macrophages (19–21). AMB and L-AmB have provided remarkable curative effects and low recurrence rates in the treatment of VL. They are also applicable for patients who experience relapse. AMB was first used to treat VL in other countries in the 1960s, and it was later introduced in China. To date, AMB has been rarely used in China, and its use in the literature was limited to antimony-resistant leishmaniasis (22–24). This might be related to its side effects. By contrast, L-AmB is readily absorbed and highly bioavailable with low rates of adverse events compared with standard AMB, and it is a front-line agent in various endemic areas (21). However, clinical data for L-AmB remain extremely limited, especially in underdeveloped areas, because of its prohibitive price. In this article, we reported the treatment of four patients with VL who received the low-dose L-AmB therapy. All four patients achieved etiological cure without severe complications.

According to the instructions of L-AmB, treatment is usually started at a dose of 0.1 mg/(kg·day) via slow intravenous infusion with a drip rate not exceeding 30 drops/min. If tolerated, this dose could be increased in 0.25–0.50mg/kg increments per day up to 1–3 mg/(kg·day) for maintenance therapy. Different treatment regimens have been introduced for patients with VL in different regions. The regimen recommended by WHO is a dose of 3–5mg/(kg·day) for 3–5 days (the cumulative dose can be up to a maximum of 15 mg/kg) or a single dose of 10 mg/kg (15). The regimen recommended by the US FDA is 3 mg/(kg·day) on days 1–5, 14, and 21, with an accumulative dose of 21 mg/kg (25). According to these recommendations, we intended to start with a low dose and maintain it at 3–5mg/(kg·day) to achieve the target cumulative therapeutic dose. However, the first patient experienced transient renal dysfunction and hypotension when the dose was quickly increased to 2 mg/(kg·day) at day 4. The therapy had to be stopped until his blood pressure stabilized and his kidney function improved. In order to complete the treatment, we subsequently adopted the low-dose therapy as described above, which proved to be equally safe and effective. To avoid the similar situation as patient 1, patient 2, 3, and 4 were also treated with low-dose L-AmB and were cured clinically without serious adverse events. A recent study indicated that empirical L-AmB therapy at a low dose [1 mg/(kg·day)] might be associated with greater medication safety and better economic benefits than high-dose treatments (26). In addition, the low-dose L-AmB therapy has been successfully applied in pulmonary fungal infection (27). Overall, this novel treatment protocol we adopted showed its significant tolerability, efficacy, and safety in the treatment of primary, recurrent, or severe VL.

In summary, the low-dose L-AmB therapy is novel and unique because of its low initial dose, cautious dose-escalation protocol, and low maintenance dose, and it proved effective and minimally toxic in patients with VL. This individualized and flexible treatment reaches the recommended cumulative dose in a safe and controlled manner. The low-dose L-AmB therapy is a novel and promising method for patients with primary, recurrent, or severe VL. However, further large prospective, multicenter, randomized controlled, trails are needed to further evaluate the clinical applicability and safety of this treatment protocol.

## Data Availability Statement

The original contributions presented in the study are included in the article/Supplementary Material, further inquiries can be directed to the corresponding authors.

## Ethics Statement

Written informed consent was obtained from the individual(s) for the publication of any potentially identifiable images or data included in this article.

## Author Contributions

XZ, DR, and WC designed research and wrote the article. XL and QH conceived the idea of the case report. HX drew the figure. WF and GL revised the article. All authors reviewed the final version of the manuscript and agreed to its submission.

## Funding

This work was supported by the National Science and Technology Projects (Project Nos. 2017ZX10203201, 2017ZX10201201, 2017ZX10202202, and 2018ZX10302206) and the National Natural Science Fund (Project No. 81700559).

## Conflict of Interest

HX was employed by the company Hugobiotech Co., Ltd. The remaining authors declare that the research was conducted in the absence of any commercial or financial relationships that could be construed as a potential conflict of interest.

## Publisher's Note

All claims expressed in this article are solely those of the authors and do not necessarily represent those of their affiliated organizations, or those of the publisher, the editors and the reviewers. Any product that may be evaluated in this article, or claim that may be made by its manufacturer, is not guaranteed or endorsed by the publisher.

## Abbreviations

VL: Visceral leishmaniasis
AMB: Amphotericin B
L-AmB: Liposomal amphotericin B
AKI: Acutekidney impairment
HLH: Hemophagocytic lymphohistiocytosis
WHO: World Health Organization
US-FDA: U.S. Food and Drug Administration
PCR test: the polymerase-chain-reaction test.
