# Supplementary figures and images for: Case Report: Use of Liposomal Amphotericin B in Low Doses in Patients With Visceral Leishmaniasis
Source: Front Med (Lausanne). 2021 Nov 17;8:766400. doi: 10.3389/fmed.2021.766400 (PMC8635719; doi:10.3389/fmed.2021.766400)

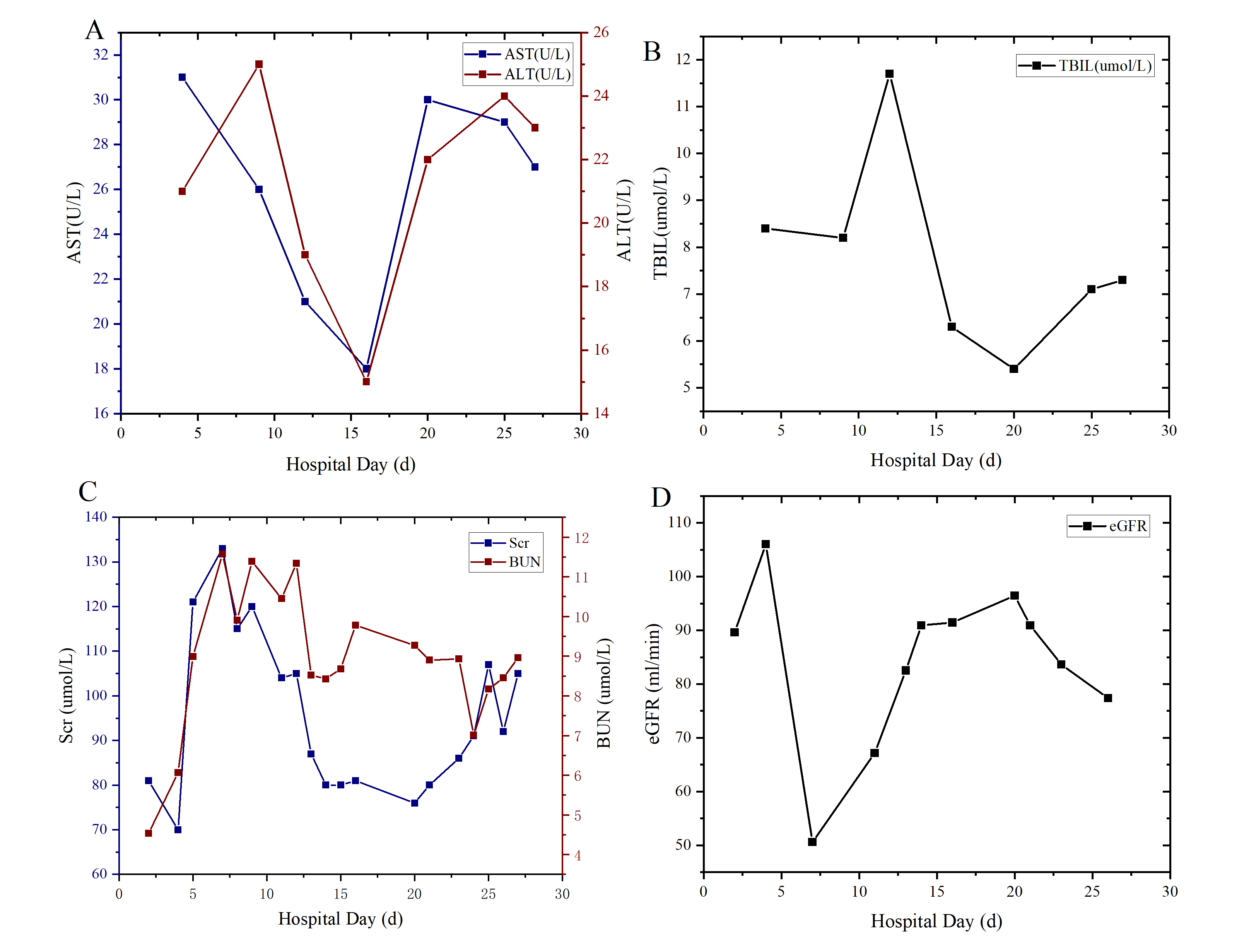

Supplement: Supplementary file 2 [file Image_1.TIF]

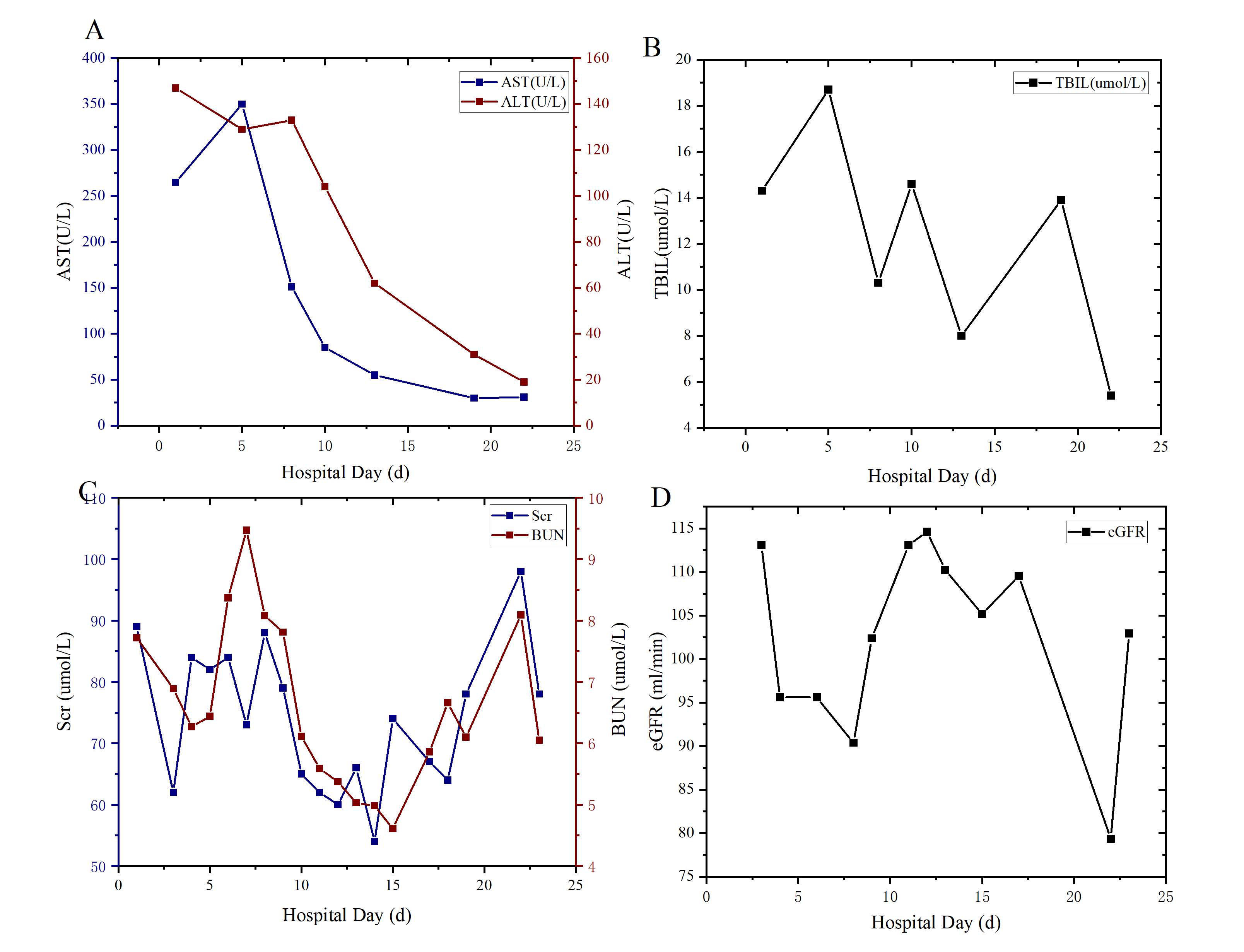

Supplement: Supplementary file 3 [file Image_2.TIF]

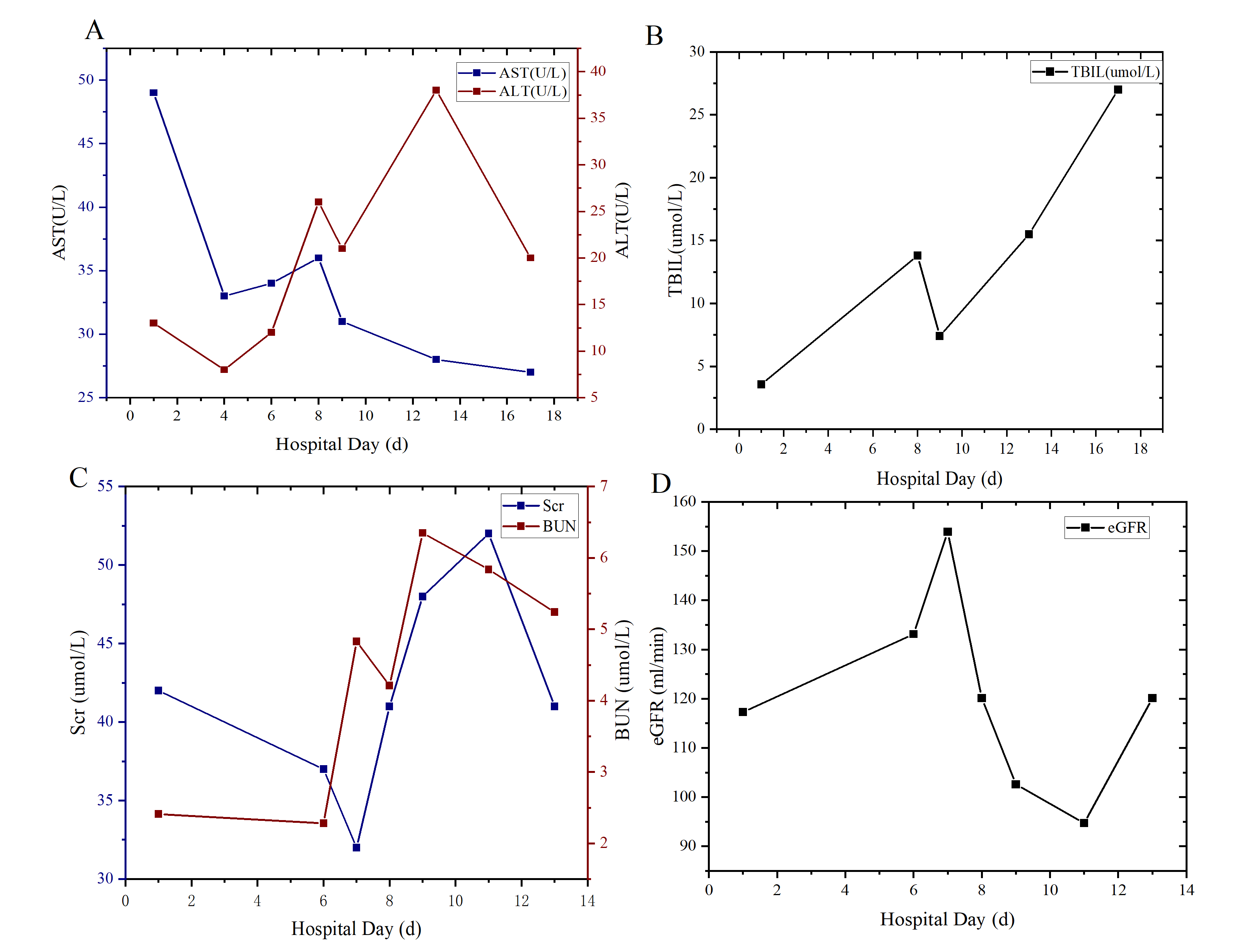

Supplement: Supplementary file 4 [file Image_3.TIF]

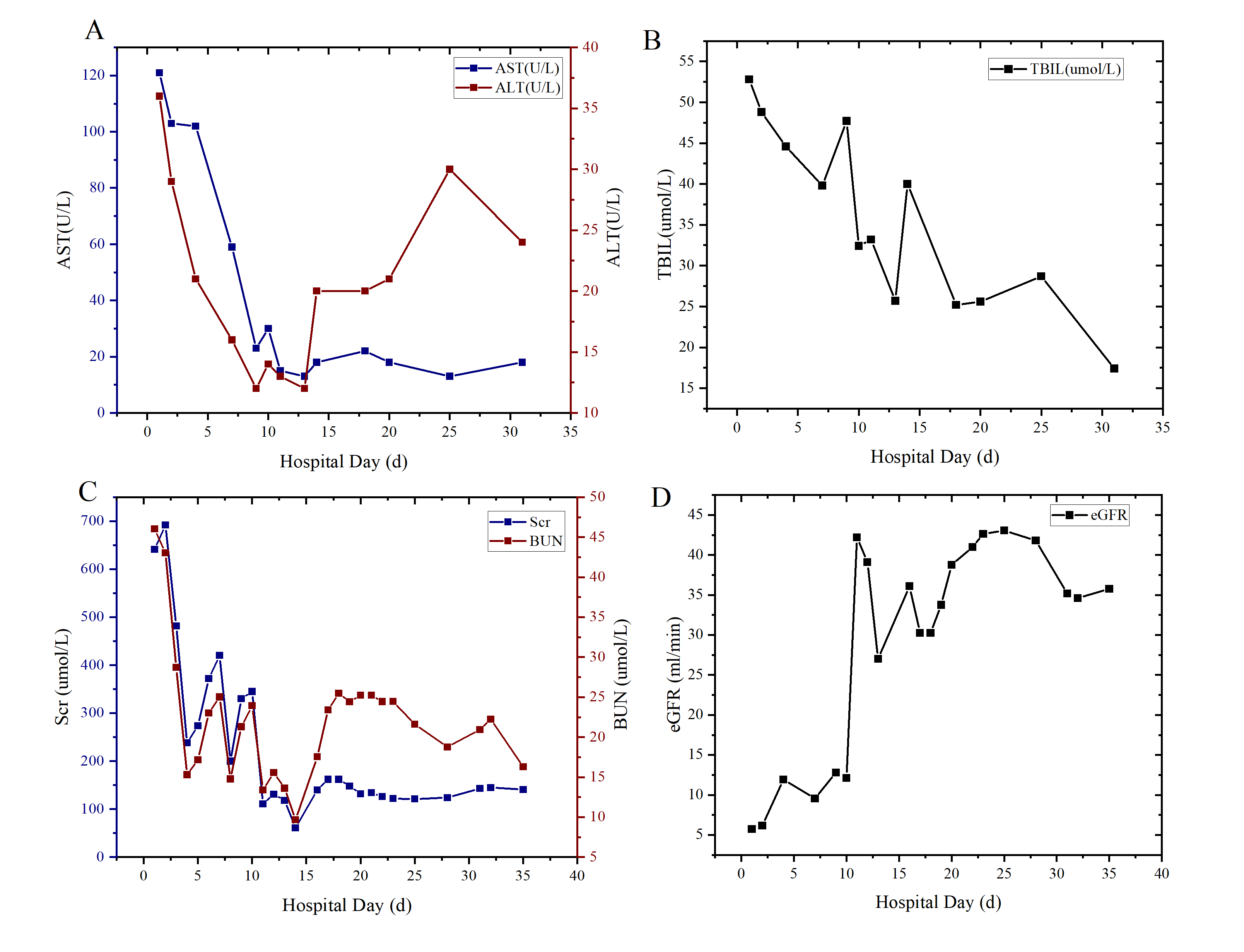

Supplement: Supplementary file 5 [file Image_4.TIF]
